# Differential Use of TLR2 and TLR9 in the Regulation of Immune Responses during the Infection with *Trypanosoma cruzi*

**DOI:** 10.1371/journal.pone.0063100

**Published:** 2013-05-01

**Authors:** Humberto D. Gravina, Lis Antonelli, Ricardo T. Gazzinelli, Catherine Ropert

**Affiliations:** 1 Centro de Pesquisas René Rachou, Fundação Oswaldo Cruz, Belo Horizonte, Minas Gerais, Brazil; 2 Departamento de Bioquímica e Imunologia, Universidade Federal de Minas Gerais, Belo Horizonte, Minas Gerais, Brazil; 3 Division of Infectious Diseases and Immunology, University of Massachusetts Medical School, Worcester, Massachusetts, Untied States of America; Karolinska Institutet, Sweden

## Abstract

Pathogens express ligands for several TLRs that may play a role in the induction or control of the inflammatory response during infection. Concerning *Trypanosoma cruzi,* the agent of Chagas disease, we have previously characterized glycosylphosphatidylinositol (GPI) anchored mucin-like glycoproteins (tGPI-mucin) and unmethylated CpG DNA sequences as TLR2 and TLR9 agonists, respectively. Here we sought to determine how these TLRs may modulate the inflammatory response in the following cell populations: F4/80^+^CD11b^+^ (macrophages), F4/80^low^CD11b^+^ (monocytes) and MHCII^+^CD11c^high^ (dendritic cells). For this purpose, TLR2^−/−^ and TLR9^−/−^ mice were infected with Y strain of *T. cruzi* and different immunological parameters were evaluated. According to our previous data, a crucial role of TLR9 was evidenced in the establishment of Th1 response, whereas TLR2 appeared to act as immunoregulator in the early stage of infection. More precisely, we demonstrated here that TLR2 was mainly used by F4/80^+^CD11b^+^ cells for the production of TNF-α. In the absence of TLR2, an increased production of IL-12/IL-23p40 and IFN-γ was noted suggesting that TLR2 negatively controls the Th1 response. In contrast, TLR9 was committed to IL-12/IL-23p40 production by MHCII^+^CD11c^high^ cells that constitute the main source of IL-12/IL-23p40 during infection. Importantly, a down-regulation of TLR9 response was observed in F4/80^+^CD11b^+^ and F4/80^low^CD11b^+^ populations that correlated with the decreased TLR9 expression level in these cells. Interestingly, these cells recovered their capacity to respond to TLR9 agonist when MHCII^+^CD11c^high^ cells were impeded from producing IL-12/IL-23p40, thereby indicating possible cross-talk between these populations. The differential use of TLR2 and TLR9 by the immune cells during the acute phase of the infection explains why TLR9**-** but not TLR2**-**deficient mice are susceptible to *T. cruzi* infection.

## Introduction

The discovery of Toll-like receptors (TLRs) has contributed to a new understanding of the complexity of the role of innate immunity in infectious diseases. These receptors have been described as the first line of defense against microbial infections by bacteria, virus, fungi and protozoa [1]–[3]. Thus far, ten TLRs have been reported in humans and 13 in mice, and are classified according to their sub-cellular localization. It is now commonly accepted that pathogens possess different TLR agonists and that activation of more than one TLR is involved in the host immune response. *In vitro*, it was found that certain TLRs can act synergistically in response to microbial stimuli in specific, non-random, combinations [4]–[6]. Generally, *in vivo*, it was reported that cooperation between TLRs induces synergistic functions. For instance, the synergy between TLR2, TLR4 and TLR9 for induction of the MyD88-dependent splenic cytokine and chemokine response has been related in a model of *Streptococcus pneumoniae* infection [7]. In another study, Bahn *et al.* presented data suggesting that the maximal induction of IL-23 and IL-17 required both TLR4 and TLR9 in lung innate responses during Gram-negative bacterial pneumonia confirming the importance of cooperation between TLRs [8].

In the case of *Trypanosoma cruzi*, the etiologic agent of Chagas disease, our group and collaborators have identified different major components from this parasite capable of activating TLRs in dendritic cells (DCs) and macrophages. More precisely, GPI-anchored mucin-like glycoproteins (tGPI-mucin) from parasite membrane were shown to initiate the inflammatory response through an activation of TLR2 [9], [10], while immunostimulatory, unmethylated CpG motifs present in *T. cruzi* genome were identified as a TLR9 agonist [11], [12]. Others TLRs, like TLR4 and TLR7, have been involved in immune response during the first stage of infection and their role reviewed in the reference [13].

As previously reported, the establishment of Th1 response is required for host resistance to *T. cruzi* infection [14], [15]. In this regard, the role of TLRs has been investigated; TLR9 was shown to be crucial in the parasitemia control and mouse survival [12], whereas TLR2 was defined as immunoregulator [16]. A possible cooperative role of both receptors during infection has been suggested in a study that evaluated the resistance to infection of TLR2/TLR9-double knockout (TLR2/TLR9^−/−^) mice [11]. The singularity of the parasitic infection model used in our study is the control of inflammatory response in the acute phase of infection [17] differing from others infections where excessive TLR signaling pathway activation leads to pathogenesis [18], [19]. In this context, a definition of the role of TLR2 and TLR9 in the most important cells for inflammatory cytokine production appeared relevant. More specifically, we have evaluated how TLRs during *T. cruzi* infection may regulate the pro-inflammatory activity of F4/80^+^CD11b^+^ considered mainly as macrophages, F4/80^low^CD11b^+^ as monocytes and monocyte-derived populations, and MHCII^+^CD11c^high^ as DCs.

We show here that TLR9 is the main receptor involved in IL-12/IL-23p40 release by DCs. Furthermore, the role of TLR9 is amplified in DCs during the infection which contrasts with the dramatically decreased capacity of the macrophage/monocyte lineage to respond to TLR9 agonist. It has been possible to establish a correlation between the activity and the expression level of TLR9 in these distinct cells. Uncommonly, TLR2 possesses a dual role during the infection. TLR2 is shown to control IL-12/IL-23p40 release by DCs and at the same time to promote TNF-α production by macrophages. The different functions of TLR2 and TLR9 observed explain the impact of their deficiency on the resistance to infection. This study reveals the level of complexity of the interactions between TLRs and immune cells.

## Materials and Methods

### Ethics Statement

The experiments with mice were performed in accordance with the guidelines of the Institutional Animal Care and Committee on Ethics of Animal Use (Comitê de Ética do Uso de Animal) from Fundação Oswaldo Cruz, protocol P-53/09-5 approved in 03/15/2010.

#### Mice

TLR2^−/−^, TLR9^−/−^ mice were generated by Dr. Shizuo Akira at Osaka University (Osaka, Japan). All the knockout mice were backcrossed with C57BL/6 for at least eight generations. All the mice, including the wild-type (C57BL/6) controls, were raised in micro-isolators in the animal room at the Instituto René Rachou, Fundação Oswaldo Cruz (Belo Horizonte, Minas Gerais, Brazil).

#### Reagents

Reagents used were obtained from Sigma-Aldrich (St. Louis, MO, USA) unless indicated otherwise. IFN-γ, TNF-α, IL-12/IL-23p40 concentrations were measured in cell culture supernatants with Duoset ELISA kits from R&D Systems Inc. (Minneapolis, MN, USA); CpG DNA (TCGACGTTTGGATCGGT) derived from *T. cruzi* genome was synthesized in a phosphorothioate backbone and purchased from the Coley pharmaceutical group (Wellesley, MA, USA).

#### Experimental infection with *T. cruzi*

Mice were infected i.p. with 100 bloodstream trypomastigote forms of the Y strain of *T. cruzi*.

#### Flow cytometric analysis and cytokine measurements in mouse splenic cells

For analysis of tissue-derived cells, spleens were first dissociated with collagenase treatment (Calbiochem, Someville, NJ, USA), followed by incubation at 37°C for 30 min, and forced through a 100 µm filter (BD Biosciences, Bedford, MA, USA). Splenic cells were centrifuged for 10 min at 700×g at 4°C, supernatants were discarded, the cell pellets re-suspended in ACK lysing buffer in order to lyse erythrocytes. The cell suspensions were stained with mAb specific for CD11c-FITC, MHC-II-APC (BD Biosciences - Pharmingen); F4/80–APC and CD11b-PECy7 (eBioscience) to phenotype splenic cells during the analyses of TLR expression, cytokine measurement, and reconstitution with macrophages. For the analysis of TLR expression, specific anti-TLR2-PE mAb was added together with antibodies against cell surface markers. Cells were permeabilized according to the kit instructions (Fix/Perm kit; BD Biosciences) for the evaluation of TLR9 expression before the addition of specific anti-TLR9-biotin (eBioscience) followed by Streptavidin PE (eBioscience). Concerning the experiments evaluating TLR expression, we used cells lacking the TLR of interest as control for antibody binding; this strategy allowed us to define the positive versus negative population in these experiments.

For the intracellular cytokine measurement, freshly isolated cells were analyzed after culture for 6 h with medium alone, LPS (100 ng/ml), CpG DNA (1 µg/ml), Pam3Cys (1 µg/ml) in the presence of brefeldin A (1 µg/ml). The permeabilized cells were staining with mAb specific for TNF-α and IL-12/IL-23p40 (PE labeled) (BD Biosciences - Pharmingen). Data were collected using a FACSCalibur (BD Biosciences - Immunocytometry Systems) with Cell Quest Pro (BD Biosciences) and analyzed with FlowJo (Tree Star) software. Alternatively, splenocytes were cultured in 24-well plates (5×10^6^ cells/well) at 37 C/5% CO_2_ and stimulated or not with LPS (1 µg/ml), Pam3Cys (1 µg/ml), and CpG DNA (1 µg/ml). Supernatants from spleen cell cultures were analyzed for TNF-α, IL-12/IL-23p40, and IFN-γ levels after 48 and 72 h, respectively.

#### Macrophage transfer experiments

TLR9^−/−^ mice were injected intraperitoneally with 10^7^ peritoneal exudate WT macrophages as described previously [20]. Four days later, mice were infected with *T. cruzi* (100 trypomastigotes form per animal). Spleens were harvested 7 days pos-infection and cell phenotype was analyzed as described in Flow cytometric analysis section.

#### Statistical Analysis

The statistical significance of the data was determined by an unpaired Student’s test and values are shown as *p<0.05 and **p<0.01.

## Results

### TLR2 and TLR9 do not Play a Redundant Role During T. cruzi Infection

**Figure 1:**
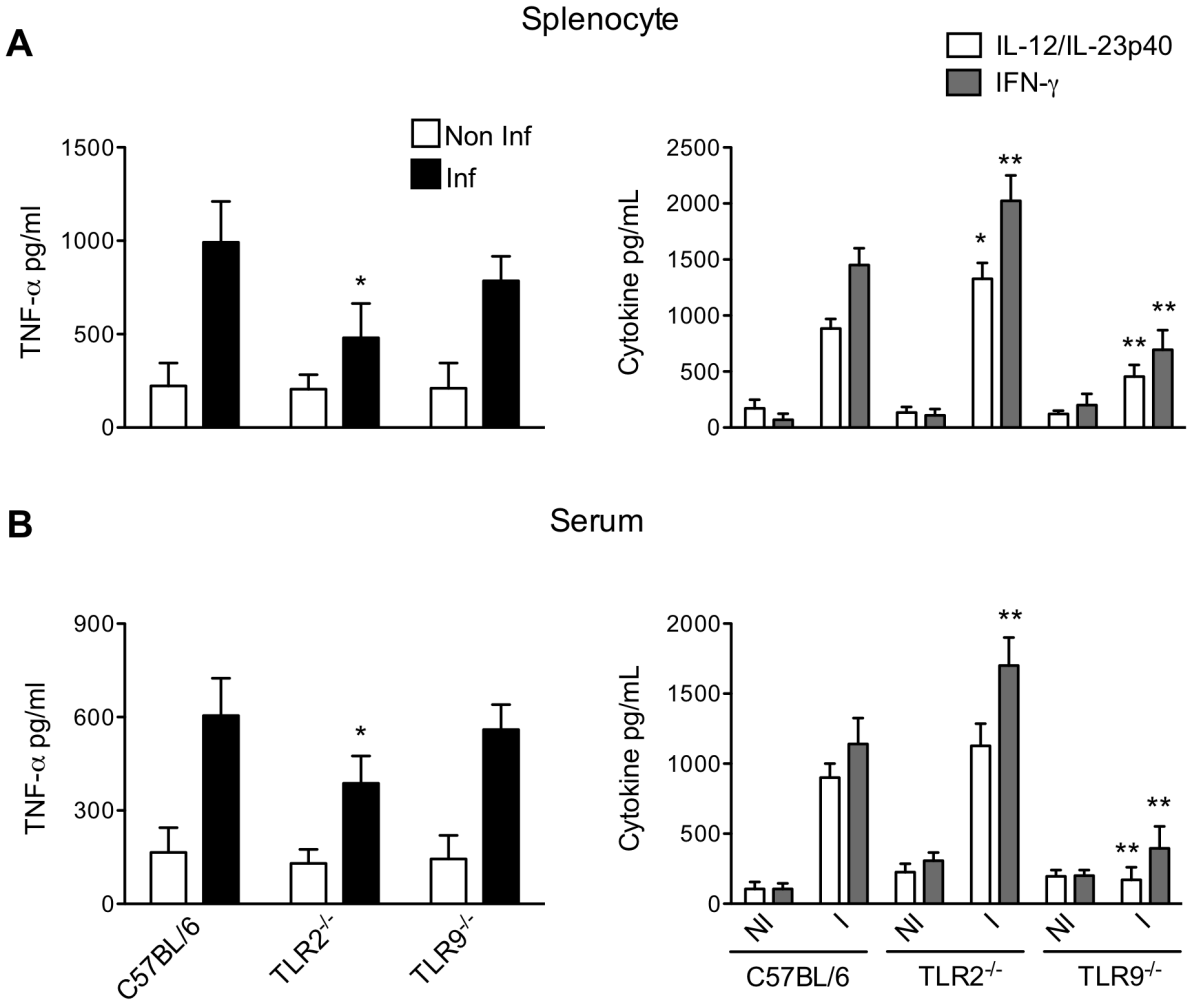
Comparison of the proinflammatory response in TLR2- and TLR9-deficient mice during the acute phase of *T.cruzi* infection. Cytokine levels in spleen cell culture (A) and serum (B) from either control (NI) or infected (I) C57BL/6 WT, TLR2^−/−^, TLR9^−/−^ mice evaluated seven days post-infection. The data represent the mean of two experiments. *p<0.05 and **p<0.01 indicate statistical significance when comparing cytokine level in serum or in splenocyte culture from knockout versus C57BL/6 WT infected mice.

As previously published, immune responses induced during infection with *T. cruzi* consist of a highly polarized type 1-cytokine response essential for host resistance [14], [15]. tGPI-mucin, one of the major glycoproteins of *T. cruzi* plasma membrane, and CpG motifs present in *T. cruzi* DNA have been identified as TLR2 and TLR9 agonists, respectively, both able to induce pro-inflammatory cytokine release in macrophages and BMDCs [10], [12]. But *in vivo*, the role of TLR2 and TLR9 are quite different during the *T. cruzi* infection. TLR2 does not affect the parasitemia and mortality, whereas TLR9 appears to be crucial for the control of parasite replication and mouse survival [12], [16]. Here we analyzed the requirement of TLR2 and TLR9 in the release of TNF-α, IL-12/IL-23p40 and IFN-γ, all present before the peak of parasitemia. As observed in Figure 1, the decrease in IL-12/IL-23p40 level correlated with the decreased in IFN-γ level in splenocyte culture or in serum from mice lacking TLR9. This confirmed that TLR9 is actively engaged in the establishment of Th1 response. Furthermore, these data allow to claim that p40 subunit detected in our assays corresponds to IL-12 and not to IL-23 since IL-23 is not efficient as IL-12 in the induction of IFN-γ [21]. By contrast, TNF-α level remained unchanged in splenocyte culture or serum from mice lacking TLR9 indicating that TLR9 did not control TNF-α production and that TNF-α was released independently on IL-12/IL-23p40 and IFN-γ levels. Furthermore, according to the data in Figure 1, TLR2 assumed a dual function in this phase of infection. In the absence of TLR2, a significant increase of IFN-γ and IL-12/IL-23p40 responses were observed confirming the regulatory role of TLR2 as previously reported [16]. At the same time, TLR2 appeared partially responsible for the TNF-α release as a reduction of about 40% of this cytokine was observed in splenocyte culture or serum from infected TLR2^−/−^ mice. Together, our data clearly underscore the differential involvement of TLR2 and TLR9 in the release of TNF-α, IL-12/IL-23p40 and IFN-γ.

### DCs and Macrophages Share Function during the Acute Phase of Infection with *T. cruzi*

**Figure 2:**
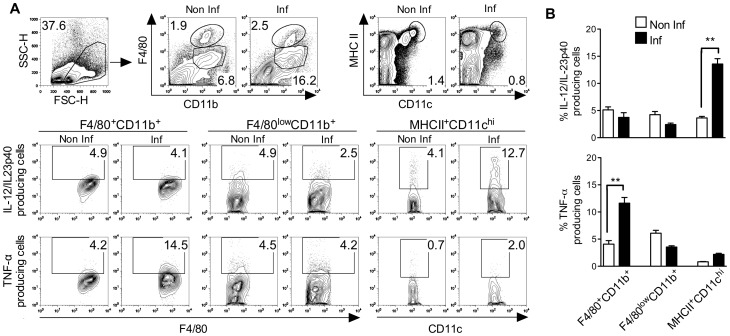
Evaluation of the capacity of F4/80^+^CD11b^+^, F4/80^low^CD11b^+^ and MHCII^+^CD11c^high^ populations to produce TNF-α and IL-12/IL-23p40 during the acute phase of *T.cruzi* infection. Splenic cells were analyzed seven days post-infection. (A) Representative flow cytometry plots showing the exclusion of debris and non-interest population of interest (FSC-H X SSC-H) and the assortment of immune cells from non-infected or infected mice. Representative flow cytometry plots showing intracellular cytokine in the different cells from non-infected or infected mice. (B) Frequencies of IL-12/IL-23p40^+^ or TNF-α^+^ splenic cells (F4/80^+^CD11b^+^, F4/80^low^CD11b^+^ or MHCII^+^CD11c^high^) (mean ± SD of four mice) isolated from non-infected or infected mice. Data are representative of two independent experiments. **p<0.01 indicates statistical significance when comparing the percentage of the same cell population from infected versus non infected mice involved in TNF-α or IL-12/IL-23p40 production.

Here we aimed to identify the cellular source of IL-12/IL-23p40 and TNF-α in the acute phase of infection. This can help to understand how different types of innate immune cells communicate with one another. In this context, the capacity of F4/80^+^CD11b^+^, F4/80^low^CD11b^+^ and MHCII^+^CD11c^high^ cells to produce cytokine (TNF-α and IL-12/IL-23p40) was evaluated seven days post-infection. Flow cytometry plots illustrate how the different populations were defined (Fig. 2A). At first, we compared the capacity of each population to produce IL-12/IL-23p40. The intracellular staining showed a significant increase of the percentage of IL-12/IL-23p40^+^MHCII^+^CD11c^high^ cells in infected mice. Indeed, about 13% of MHCII^+^CD11c^high^ cells classified as mature DCs (DCs) were committed to IL-12/IL-23p40 production (Fig. 2B). By contrast, no increase of IL-12/IL-23p40^+^F4/80^+^CD11b^+^ or IL-12/IL-23p40^+^F4/80^low^CD11b^+^ cells was observed during the infection suggesting that in our model these populations did not constitute a source of IL-12/IL-23p40. Interestingly, splenic MHCII^+^CD11c^high^ population identified as the main producer of IL-12/IL-23p40 was not involved in TNF-α production during *T. cruzi* infection. As shown in Figure 2B, we found that macrophages were implicated in TNF-α production in the acute phase of infection since an increased percentage (∼13%) of TNF-α^+^F4/80^+^CD11b^+^ population was observed in spleen from infected mice. In addition, we showed that the F4/80^low^CD11b^+^ population did not contribute to TNF-α release. Thus, we conclude that during *T. cruzi* infection the cellular sources of TNF-α and IL-12/IL-23p40 are different and defined as macrophages and DCs, respectively.

### Differential Involvement of TLR9 and TLR2 in IL-12/IL-23p40 and TNF-α Release by DCs and Macrophages during *T. cruzi* Infection

**Figure 3:**
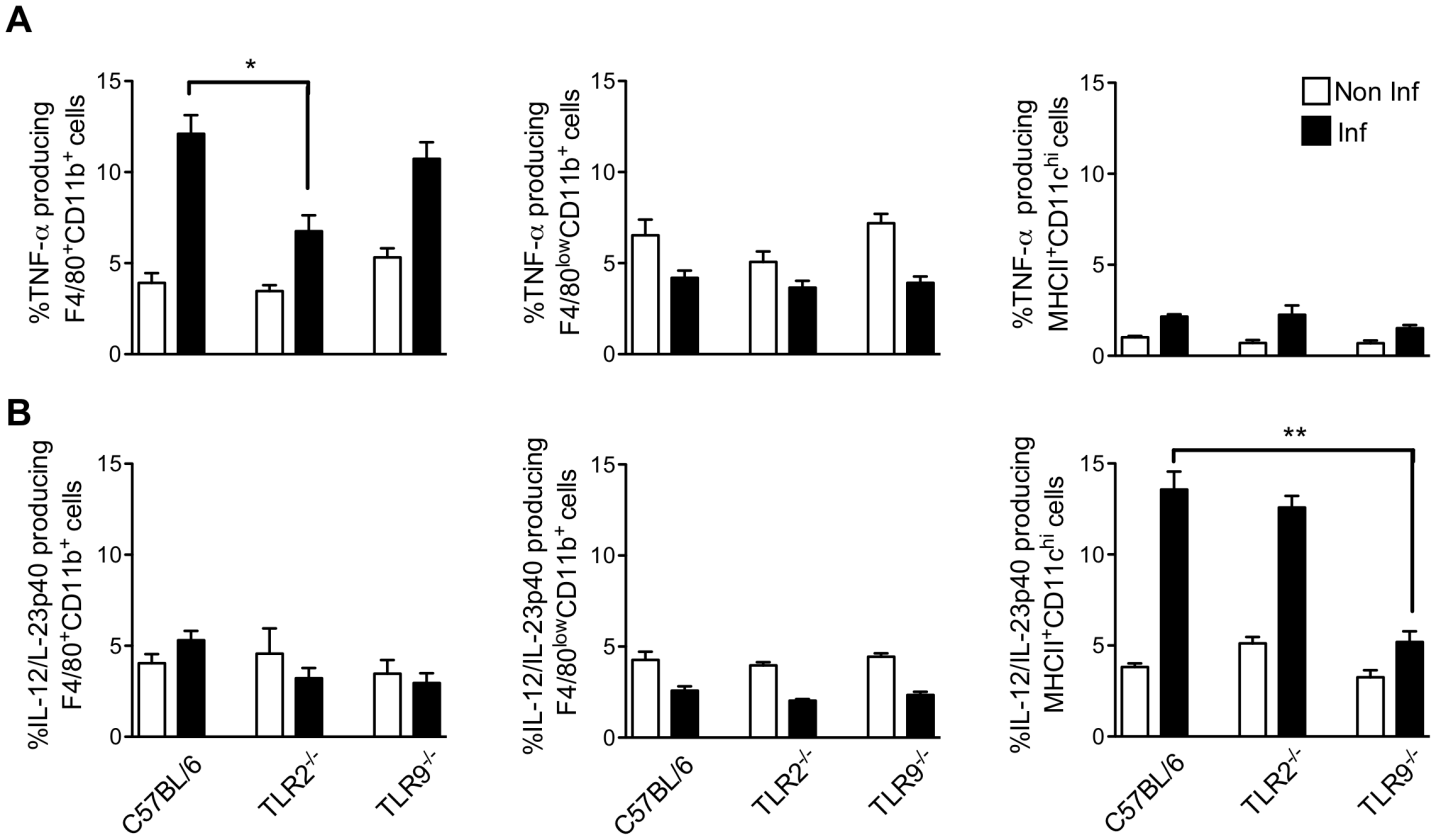
Role of TLR2 and TLR9 in TNF-α and IL-12/IL-23p40 production by F4/80^+^CD11b^+^, F4/80^low^CD11b^+^ and MHCII^+^CD11c^high^ populations from mice acutely infected with *T.cruzi*. Intracellular cytokine was analyzed by flow cytometry in spleen from C57BL/6 WT, TLR2^−/−^, and TLR9^−/−^ mice seven days post-infection. Frequencies of splenic TNF-α^+^ (A) or IL-12/IL-23p40^+^ (B) cells (mean ± SD of four mice) isolated from infected and non-infected mice. Data are representative of two independent experiments. *p<0.05 and **p<0.01 indicate statistical significance when comparing the percentage of the same cell population from knockout versus C57BL/6 WT infected mice involved in TNF-α or IL-12/IL-23p40 production.

According to the previous data, TNF-α and IL-12/IL-23p40 are produced by different cells and their production depends on different TLR activation during infection. Here, we aimed to recapitulate such findings by evaluating the intrinsic TLR activity in the different cells involved in cytokine production. First, we assessed the impact of the absence of TLR2 or TLR9 on the percentage of TNF-α^+^ cells during the infection. As observed in Figure 3A, it appeared that the ability of F4/80^+^CD11b^+^ cells to produce TNF-α was significantly reduced in the absence of TLR2. In fact, infected TLR2^−/−^ mice had ∼40% less TNF-α^+^F4/80^+^CD11b^+^ compared with infected WT mice. By contrast, the lack of TLR9 had no impact on the capacity of these cells to produce TNF-α.

When we investigated the role of TLR in the production of IL-12/IL-23p40 by MHCII^+^CD11c^high^ cells (Fig. 3B), we noted that the capacity of this cell population to produce this cytokine in response to *T. cruzi* infection was highly impaired in cells lacking TLR9. Importantly, the absence of TLR2 did not affect the number of IL-12/IL-23p40^+^MHCII^+^CD11c^high^ cells. In addition, neither F4/80^+^CD11b^+^ nor F4/80^low^CD11b^+^ cells produced IL-12/IL-23p40 to compensate the decreased number of IL-12/IL-23p40^+^MHCII^+^CD11c^high^ observed in TLR9^−/−^ mice. In summary, it appears that TLR9 and TLR2 are respectively critical receptors for IL-12/IL-23p40 production by DCs and TNF-α release by macrophages.

### *T. cruzi* Induces Priming of TLR9 Responses in Splenic Cell

**Figure 4:**
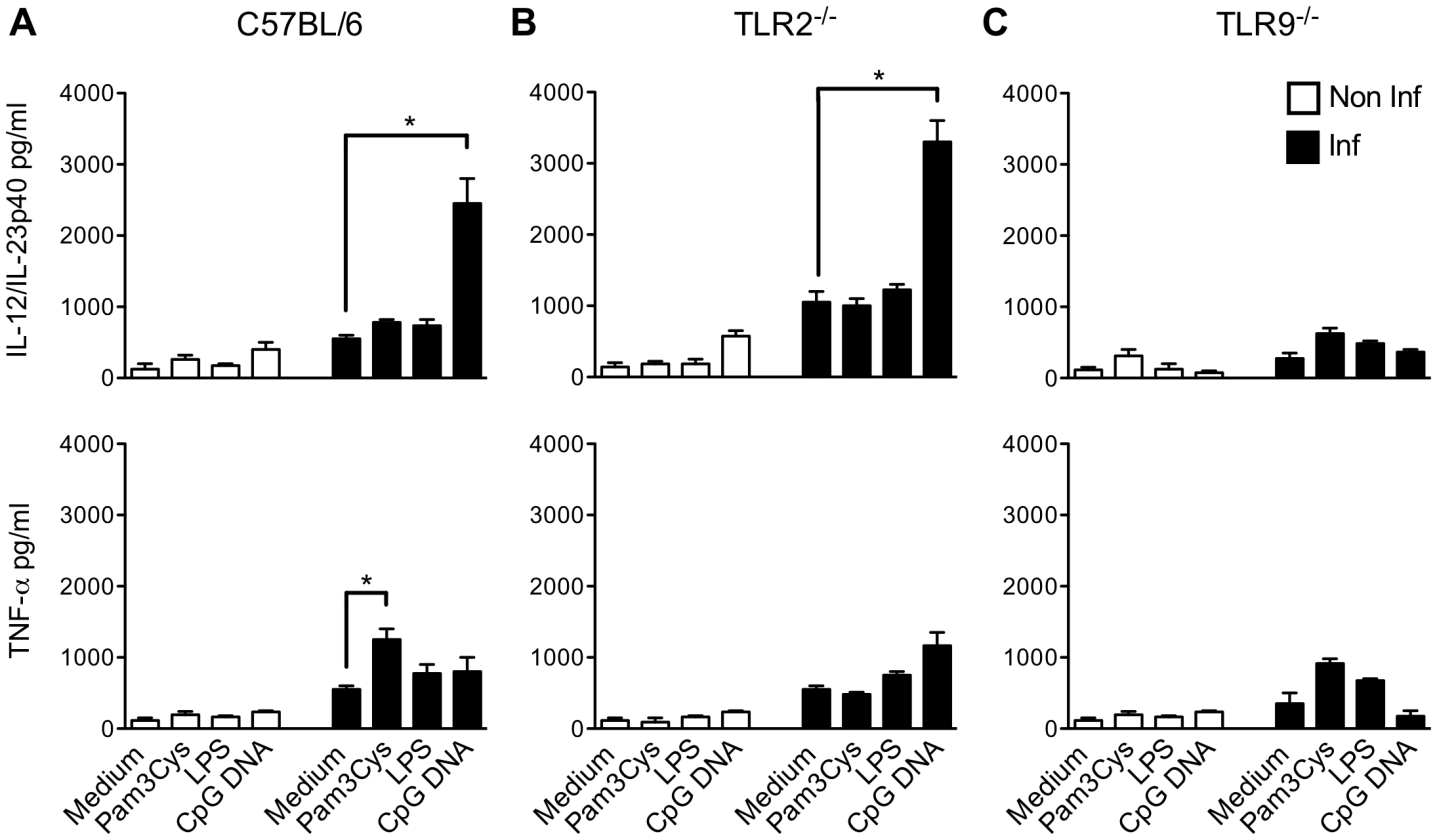
Evaluation of cytokine production by splenic cells from *T.cruzi* infected mice in response to TLR agonists. Cytokine levels in spleen cell culture stimulated or not with LPS (1 µg/ml), Pam3Cys (1 µg/ml) or CpG DNA (1 µg/ml) from either control (non-infected) or infected C57BL/6 WT, TLR2^−/−^, and TLR9^−/−^ mice seven days post-infection. Supernatants from spleen cell cultures from C57BL/6 WT (A), TLR2^−/−^ (B) and TLR9^−/−^ mice (C) were analyzed for IL-12/IL-23p40 or TNF-α after 48h. Data are representative of two independent experiments. *p<0.05 indicates statistical significance when comparing cytokine release by spleen cells stimulated or not with TLR agonist in a same group (infected or not infected mice).

As demonstrated above, during *T. cruzi* infection IL-12/IL-23p40 release was TLR9-dependent, whereas TLR2 was involved in TNF-α release. We then investigated the quantitative involvement of TLR9 and TLR2 in the establishment of Th1 response (Fig. 4). For this purpose, we compared the capacity of splenocytes from infected and non-infected WT, TLR2^−/−^ and TLR9^−/−^ mice to produce cytokines in response to Pam3Cys and CpG DNA. As shown here, TLR responses were differentially modulated during the infection: responses to TLR2 agonist (Pam3Cys) were moderately boosted (1.5 fold increase) during *T. cruzi* infection; whereas, the infection induced a priming of the TLR9, leading to dramatically increased production of IL-12/IL-23p40 (6 fold) when exposed to TLR9 ligand (CpG DNA) (Fig. 4A). Importantly, the same was not verified for TNF-α production, reinforcing the idea that TLR9 was mainly involved in IL-12/IL-23p40 release. In the absence of TLR2, the production of IL-12/IL-23p40 by splenic cells in response to CpG DNA was higher (Fig. 4B), thus underscoring the immunoregulatory role of TLR2. The capacity of TLR2 agonist to induce IL-12/IL-23p40 remained low even in the absence of TLR9 (Fig. 4C). This probably explained the susceptibility of TLR9^−/−^ mice during the infection since TLR2 cannot assume IL-12/IL-23p40 production required for mice survival in such situation. When taken together, our data confirmed the crucial role of TLR9 in the establishment and amplification of Th1 response through IL-12/IL-23p40 release.

### *T. cruzi* Causes a Decreased Response to TLR9 Agonist in Macrophage/monocyte Lineage

**Figure 5:**
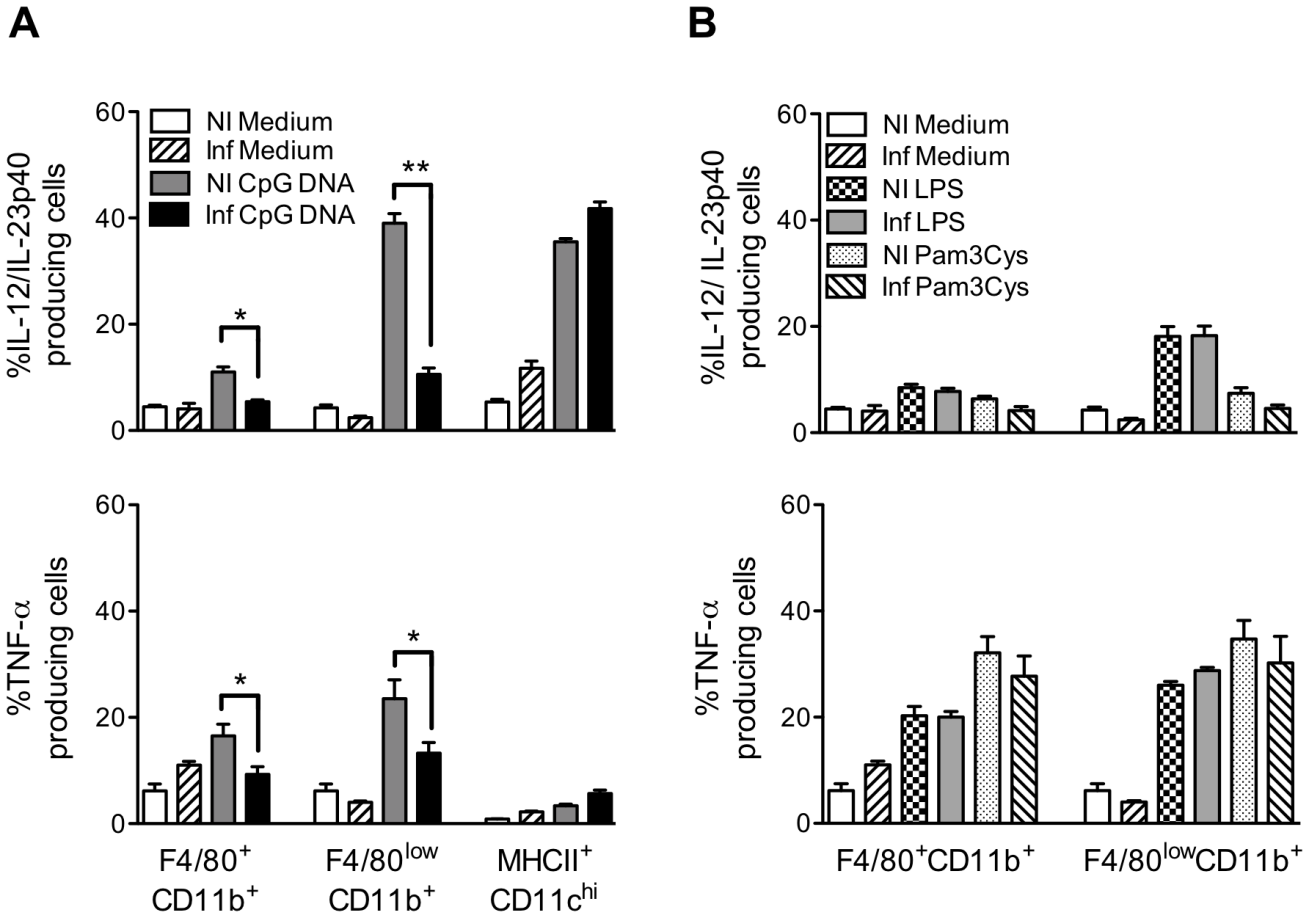
Comparison of the capacity of F4/80^+^CD11b^+^, F4/80^low^CD11b^+^ and MHCII^+^CD11c^high^ populations to respond to TLR9 agonist during acute phase of *T.cruzi* infection. Intracellular TNF-α and IL-12/IL-23p40 were analyzed by flow cytometry in spleen from C57BL/6 mice seven days post-infection. Splenic cells were cultured in medium alone, with CpG DNA (1 µg/ml), LPS (1 µg/ml) or Pam3Cys (1 µg/ml). (A) Frequencies of splenic TNF-α^+^ or IL-12/IL-23p40^+^ cells (F4/80^+^CD11b^+^, F4/80^low^CD11b^+^ and MHCII^+^CD11c^high^) after stimulation with CpG DNA (1 µg/ml) (mean ± SD of four mice) or (B) frequencies of splenic TNF-α^+^ or IL-12/IL-23p40^+^ cells (F4/80^+^CD11b^+^ and F4/80^low^CD11b^+^) stimulated with LPS (1 µg/ml) or Pam3Cys (1 µg/ml) (mean ± SD of four mice) isolated from infected and non-infected mice. Data are representative of three independent experiments. *p<0.05 and **p<0.01 indicate statistical significance when comparing the percentage of the same cell population from infected or not infected mice cultured in the same conditions.

Here we sought to define the cell populations involved in TLR9 priming. In Figure 5A, we compared the capacity of F4/80^+^CD11b^+^, F4/80^low^CD11b^+^ and MHCII^+^CD11c^high^ cells to produce cytokine when stimulated with CpG DNA. The impact of TLR9 agonist on the capacity of DC population to produce IL-12/IL-23p40 is dramatic, since about 40% of MHCII^+^CD11c^high^ cells were committed to IL-12/IL-23p40 production after CpG DNA addition. The most unexpected results came from the dramatic reduction of the capacity of F4/80^+^CD11b^+^ and F4/80^low^CD11b^+^ populations to respond to TLR9 agonist during the infection. Stimulation with CpG DNA induced a decrease of TNF-α and IL-12/IL-23p40 production by F4/80^+^CD11b^+^ and F4/80^low^CD11b^+^ cells from infected mice when compared with the same population from non-infected mice (Fig. 5A). The percentage of F4/80^low^CD11b^+^ cells able to respond to TLR9 agonist after infection was reduced by ∼70%. When we performed the experiments infecting TLR2^−/−^ mice, the same phenomenon was observed excluding a role of TLR2 in the modulation of TLR9 response in these cells (data not shown). Importantly, the percentage of F4/80^+^CD11b^+^ and F4/80^low^CD11b^+^ cells capable to respond to Pam3Cys or LPS was unchanged during the infection (Fig. 5B). This led us to conclude that the reduction in TLR9 response observed was singular and did not concern TLR responses other than TLR9-mediated ones in macrophage/monocyte lineage. According to our data, it appears that TLR9 pathway activation is differentially modulated in DC, macrophage and monocyte lineage and in the case of DCs may constitute a marker of inflammatory state.

### Evaluation of the Modulation of TLR9 and TLR2 Expression on DCs and Macrophage/monocyte Lineage during *T. cruzi* Infection

**Figure 6:**
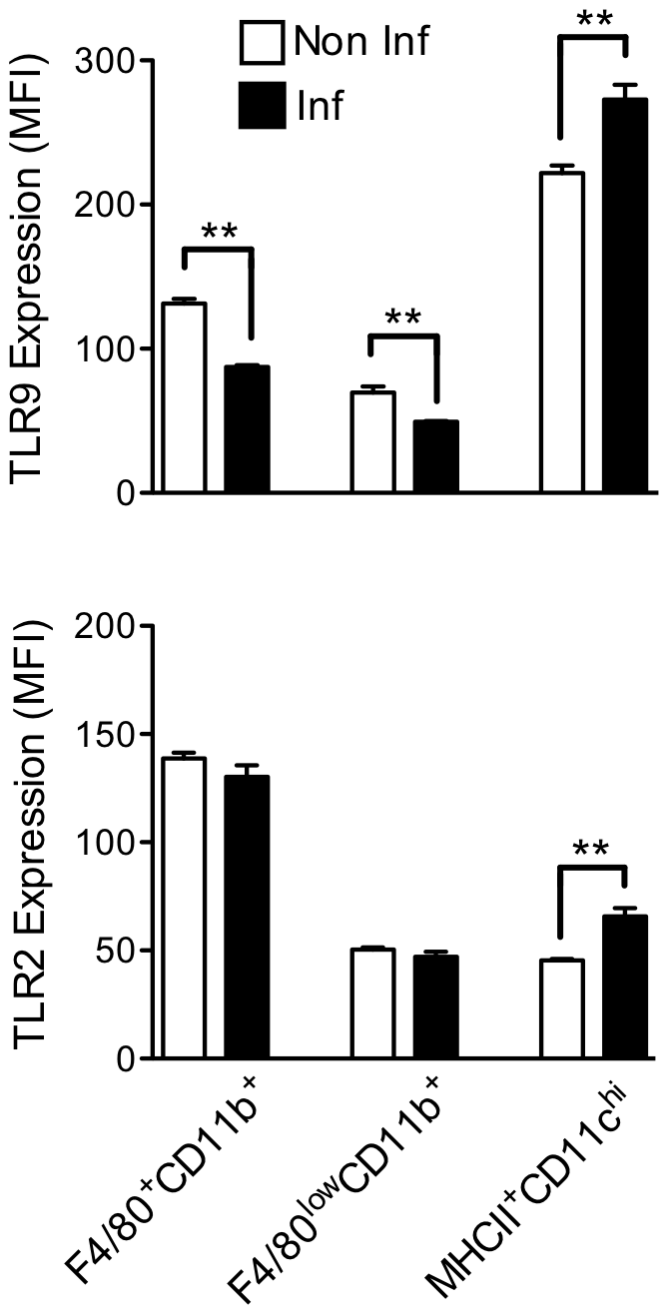
Expression level of TLR9 and TLR2 in spleen cells during the acute phase of*T. cruzi* infection. The mean fluorescent intensity (MFI) obtained by flow cytometry indicates the expression level of TLR9 and TLR2 in F4/80^+^CD11b^+^, F4/80^low^CD11b^+^ and MHCII^+^CD11c^high^ from infected or non-infected C57BL/6 WT mice seven days post-infection. The data represent the mean of four mice (mean ± SD). **p<0.01 indicates statistical significance when comparing the MFI of the same cell population from infected versus non-infected C57BL/6 WT mice.

As presented above, a decreased capacity of macrophage/monocyte lineage to respond to TLR9 agonist was observed during the acute phase of infection. One mechanism explored here to explain such phenomenon involved the modulation of TLR expression. We compared TLR9 expression on F4/80^+^CD11b^+^, F4/80^low^CD11b^+^ and MHCII^+^CD11c^high^ cells from infected and non-infected mice. As shown in Figure 6, the increase of TLR9 expression observed in MHCII^+^CD11c^high^ cells contrasted with the decreased expression of this receptor in F4/80^+^CD11b^+^ and F4/80^low^CD11b^+^ cells during infection. Importantly, TLR9 expression was similar in MHCII^+^CD11c^high^ cells from WT and TLR2^−/−^ mice (data not shown) indicating that TLR2 did not affect the expression level of TLR9. Concerning TLR2, the expression of this receptor was not significantly affected in F4/80^+^CD11b^+^ and F4/80^low^CD11b^+^ cells in the early phase of infection. In MHCII^+^CD11c^high^ cells, an increased of TLR2 expression was noted, reinforcing the hypothesis that TLR2 plays a role in these cells during infection. In conclusion, here we showed that TLR2 and TLR9 expression are differently regulated in DC, macrophage and monocyte lineage during the infection. These data agree with the capacity of the different cell populations to respond to distinct TLR agonists.

### Macrophages Recover TLR9 Response in the Absence of DC Inflammatory Activity during *T. cruzi* Infection

**Figure 7:**
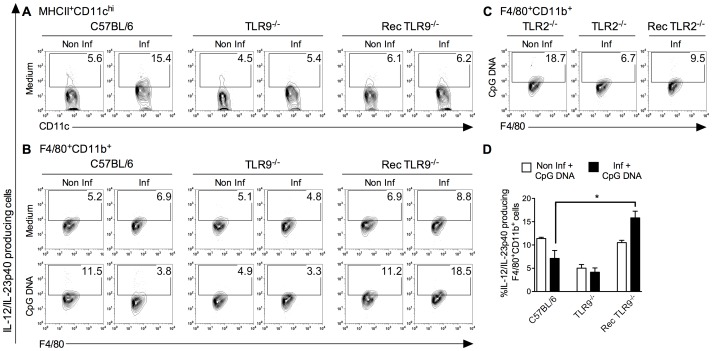
Adoptive transfer of WT macrophages in TLR9^−/−^ mice allow normal TLR response of F4/80^+^CD11b^+^ cells after *T.cruzi* infection. Representative flow cytometry plots showing (A) IL-12/IL-23p40^+^MHCII^+^CD11c^high^ and (B, C) IL-12/IL-23p40^+^F4/80^+^CD11b^+^ cells, stimulated or not with CpG DNA (1 µg/ml), from non-infected or infected C57BL/6 WT, TLR9^−/−^, TLR2^−/−^ and TLR9^−/−^ or TLR2^−/−^ mice that received WT macrophages (Rec TLR9^−/−^ or Rec TLR2^−/−^ mice). (D) Frequencies of IL-12/IL-23p40^+^F4/80^+^CD11b^+^ cells stimulated with CpG DNA (mean ± SD of four mice) isolated from non-infected or infected C57BL/6 WT, TLR9^−/−^, and Rec TLR9^−/−^ mice. **p<0.01 indicates statistical significance when compared the percentage IL-12/IL-23p40^+^F4/80^+^CD11b^+^ cells after stimulation with CpG DNA in infected C57BL/6 WT or Rec TLR9^−/−^ mice.

We hypothesized that the control of TLR9 responses in macrophage/monocyte lineage might involve inflammatory microenvironment and, more specifically, DC activation. In this context, purified WT macrophages (>90% F4/80^+^) were adoptively transferred to TLR9^−/−^ mice (Rec TLR9^−/−^ mice), whose DC inflammatory activity was considerably reduced and five days later the animals were infected. First, we confirmed that MHCII^+^CD11c^high^ cells remained incapable to produce IL-12/IL-23p40 during *T. cruzi* infection in Rec TLR9^−/−^ mice (Fig. 7A). Then, we sought to determine whether the absence of IL-12/IL-23p40 production by MHCII^+^CD11c^high^ cells might allow F4/80^+^CD11b^+^ cells to respond to TLR9 agonist. For this purpose, we compared the capacity of F4/80^+^CD11b^+^ cells from WT and from Rec TLR9^−/−^ mice to produce IL-12/IL-23p40 after CpG DNA stimulation during infection. While a decrease of TLR9 response was observed in F4/80^+^CD11b^+^ cells from infected WT mice, WT F4/80^+^CD11b^+^ cells that were transferred to TLR9^−/−^ mice showed a significant ability to produce cytokine in response to CpG DNA during *T. cruzi* infection. About 18% of F4/80^+^CD11b^+^ cells from Rec TLR9^−/−^ mice were committed in IL-12/IL-23p40 production after CpG DNA addition during the infection versus 11% in non-infected Rec TLR9^−/−^ mice (Fig. 7B and D). Thus, these data indicate a recovery of the capacity of WT F4/80^+^CD11b^+^ cells to induce IL-12/IL-23p40 after stimulation with CpG DNA when transferred to TLR9^−/−^ mice. Importantly, when the same transfer of WT F4/80^+^CD11b^+^ cells was performed in TLR2^−/−^ mice, which are characterized by a high pro-inflammatory activity of DCs, the TLR9 response by WT F4/80^+^CD11b^+^ cells was not restored (Fig. 7C). According to our data, a correlation may be established between DC inflammatory state and capacity of macrophage to respond to TLR9 agonist during the acute phase of *T. cruzi* infection.

## Discussion

Most pathogens express ligands for different TLRs. The simultaneous activation of two or more TLRs represents the likely situation during host-cell microbe interactions that contributes to the complexity of the host response [22]. Nevertheless, TLRs must be tightly controlled because excessive activation can contribute to pathogenesis [18], [19] and various mechanisms of negative TLR regulation have been evidenced [23]. During the acute stage of *T. cruzi* infection, different TLRs are triggered to combat the infection but without damages to the host [10]–[12], [16] by a rapid control of the strong inflammatory response [17]. In this context, it was interesting to investigate how TLR2 and TLR9 influence the balance pro-inflammatory/anti-inflammatory responses.

According to our data, the establishment of Th1 response depends on TLR9, but not on TLR2, which corroborates with evidence identifying the involvement of TLR9 in controlling parasitemia and survival during primary infection with *T. cruzi*. We have provided experimental evidence that DC population constitutes the main source of IL-12/IL-23p40 production in a TLR9-dependent and TLR2-independent way. Our data indicate that in the absence of TLR9, TLR2 is unable to assume a role in IL-12/IL-23p40 production. While TLR9 acts fundamentally on DC inflammatory activity, TLR2 appears to assume different functions depending on the cell type, acting as immunoregulator in DCs and producer of TNF-α in macrophages. As reported above, TLR2 is not associated with susceptibility to infection that contrasts with the role that TNF-α plays in host resistance to *T. cruzi* [24], [25]. One explanation may be the involvement of other receptors than TLR2 in TNF-α production. Nucleotide-binding oligomerization domain (Nod)-like receptors have also been identified as important in TNF-α release by BMMCs exposed to *T. cruzi* [26].

Importantly, a decreased capacity of macrophage/monocyte population to respond to TLR9 agonist is observed that contrasts with the increased responsiveness of DCs to the same TLR ligand. Such discrepancies between cells have been previously observed in different models. Pompei *et al.* have defined that DCs are more efficient in engaging TLR9 and initiating transcription of *IL-12* gene, when compared to macrophages in a model of *Mycobacterium tuberculosis* [27]. Similarly, during the infection with *Toxoplasma gondii,* the TLR adaptor (MyD88) seems to be important for DC but not for macrophage activation evidencing the discrepancies in the use of TLRs in different cell subsets [8]. The decreased response to TLR9 observed in macrophage/monocyte population during *T. cruzi* infection might be explained by the involvement of negative regulatory mechanisms. However, the participation of MyD88s (a splice variant of MyD88), a dominant-negative inhibitor of TLR signaling pathways [29] or IRAK-M mainly expressed in macrophages/monocytes that negatively regulates the TLR pathway associated with IRAK-1 [30] or the targeted degradation of Mal [31] have to be excluded since normal responses to TLR2 agonist, that require MyD88, Mal and IRAK-1, are observed in monocytes and macrophages during *T. cruzi* infection. According to our data, the decreased response to TLR9 agonist correlates with a reduced expression of TLR9 in macrophage/monocyte population. In parallel, DC priming of TLR9 responses is associated with an increased expression of TLR9. It has been proposed in different experimental infections that IFN-γ release may increase TLR expression and also primes cells to TLR responses [32]. It is likely to be the case in our infection model. In conclusion, TLR9 expression constitutes an important factor to be modulated in the different cell populations in order to control the inflammatory response.

Furthermore, the inflammatory response may be modulated directly through TLR2 and we were among the first groups to support this idea [16]. In our study, the absence of TLR2 increased the level of IL-12/IL-23p40 without augmenting the number of cells that produced IL-12/IL-23p40 (mainly DCs) suggesting that TLR2 interfered directly with the capacity of DCs to produce IL-12/IL-23p40. The immunoregulatory role of TLR2 has also been demonstrated in the *Mycobacterium tuberculosis* infection model where the absence of TLR2 led to an increased mortality due to uncontrolled inflammatory reaction [33]. In another model, it has been reported that pretreatment of keratinocytes with *S. epidermidis* lipoteichoic acid (LTA), another TLR2 agonist, prevented TLR3-induced production of cytokine [34]. Importantly, we have verified that TLR2 does not affect the level of TLR9 expression. In this context, the manipulation of signaling pathway, MAPKs and transcription factors activation in DCs represents the more likely hypothesis to explain the immunomodulatory effect of TLR2 during *T. cruzi* infection. Indeed, this is the hypothesis frequently encountered in the literature [35]–[38]. It is not excluded that Mal, a protein adapter used by TLR2 is the key downstream regulator for dictating the balance of pro-inflammatory versus anti-inflammatory gene after triggering TLR2 as suggested by Mellett *et al*. [39].

In addition to cell recruitment, cell-cell communication is a vital part of innate immunity. We have explored this aspect by evaluating the impact of inflammatory DC on TLR9 responses in macrophage. Due to its inherent plasticity macrophages are directly influenced by the inflammatory microenvironment. These cells have a plastic gene expression phenotype that changes depending on the type, concentration and longevity of exposure to the stimuli [40], [41]. In our model we verified this hypothesis: in the absence of IL-12/IL-23p40 production by DCs, macrophages recovered their capacity to respond to TLR9 agonist. We suggest here that modulation of TLR9 responses in macrophages/monocyte cells may be controlled by DC inflammatory activity.

**Figure 8:**
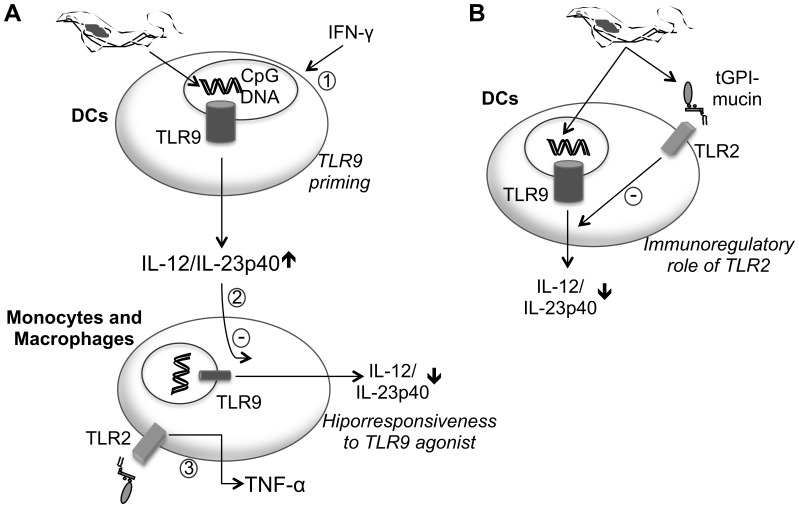
Schematic representation of the complementary effect of TLR2 and TLR9 activation during *T.cruzi* infection. A) The early release of IFN-γ induces an increase of TLR9 expression in DC and primes cells to TLR9 response (1). The high levels of IL-12/IL-23p40 secreted by DCs down-regulate the TLR9 responses of monocytes/macrophages by modulating the TLR9 expression (2). On the other hand, TLR2 is used by macrophage population to produce TNF-α (3). B) In DCs, TLR2 regulates negatively TLR9-dependent IL-12/IL-23p40 production by modulating signaling pathway.

In this paper, we sought to decode the multiple receptor interactions in order to understand the role of TLR2 and TLR9 in the modulation of the inflammatory host response during *T. cruzi* infection. According to the model shown in Figure 8, the combination of TLR2 and TLR9 can result in complementary or antagonistic effects that modulate innate immunity. As presented in Figure 8A, we propose different mechanisms to explain the modulation of TLR9 responses in immune cells. In Figure 8B, we define the role of TLR2 in the down-regulation of Th1 response during *T. cruzi* infection.

## References

[1] Takeuchi O and Akira S (2010) Pattern recognition receptors and inflammation. Cell 140(6), 805–820. 10.1016/j.cell.2010.01.022 20303872

[2] Gazzinelli RT and Denkers EY (2006) Protozoan encounters with Toll-like receptor signalling pathways: implications for host parasitism. Nat Rev Immunol 6(12), 895–906. 10.1038/nri1978 17110955

[3] Kopp E and Medzhitov R (2003) Recognition of microbial infection by Toll-like receptors. Curr Opin Immunol 15(3), 335–401. 10.1016/s0952-7915(03)00080-3 12900270

[4] Ouyang X, Negishi H, Takeda R, Fujita Y, Taniguchi T, et al.. (2007) Cooperation between MyD88 and TRIF pathways in TLR synergy via IRF5 activation. Biochem Biophys Res Commun 354(4), 1045–51. 10.1016/j.bbrc.2007.01.090 17275788

[5] Krummen M, Balkow S, Shen L, Heinz S, Loquai C, et al.. (2010) Release of IL-12 by dendritic cells activated by TLR ligation is dependent on MyD88 signaling, whereas TRIF signaling is indispensable for TLR synergy. J Leukoc Biol 88(1), 189–99. 10.1189/jlb.0408228 20360404

[6] Mäkelä SM, Strengell M, Pietilä TE, Osterlund P, Julkunen I (2009) Multiple signaling pathways contribute to synergistic TLR ligand-dependent cytokine gene expression in human monocyte-derived macrophages and dendritic cells. J Leukoc Biol 85(4), 664–72. 10.1189/jlb.0808503 19164128

[7] Lee KS, Scanga CA, Bachelder EM, Chen Q, Snapper CM (2007) TLR2 synergizes with both TLR4 and TLR9 for induction of the MyD88-dependent splenic cytokine and chemokine response to *Streptococcus pneumoniae*. Cell Immunol 245(2), 103–110. 10.1016/j.cellimm.2007.04.003 PMC2691573 17521621

[8] Bhan U, Ballinger MN, Zeng X, Newstead MJ, Cornicelli MD, et al.. (2010) Cooperative interactions between TLR4 and TLR9 regulate interleukin 23 and 17 production in a murine model of gram negative bacterial pneumonia. PLoS One 5(3), e9896. 10.1371/journal.pone.0009896 PMC2845620 20360853

[9] Almeida IC, Camargo MM, Procopio DO, Silva LS, Mehlert A, et al. (1999) Highly purified glycosylphosphatidylinositols from *Trypanosoma cruzi* are potent proinflammatory agents. EMBO J 19(7), 1476–1485. 10.1093/emboj/19.7.1476 PMC310217 10747016

[10] Campos MA, Almeida IC, Takeuchi O, Akira S, Valente EP, et al.. (2001) Activation of Toll-like receptor-2 by glycosylphosphatidylinositol anchors from a protozoan parasite. J Immunol 167(1), 416–423. 10.4049/jimmunol.167.1.416 11418678

[11] Bafica A, Santiago HC, Goldszmid R, Ropert C, Gazzinelli RT, et al. (2006) Cutting edge: TLR9 and TLR2 signaling together account for MyD88-dependent control of parasitemia in *Trypanosoma cruzi* infection. J Immunol 177(6), 3515–3519. 10.4049/jimmunol.177.6.3515 16951309

[12] Bartholomeu DC, Ropert C, Melo MB, Parroche P, Junqueira CF, et al. (2008) Recruitment and endo-lysosomal activation of TLR9 in dendritic cells infected with *Trypanosoma cruzi*. J Immunol 181(2), 1333–1344. 10.4049/jimmunol.181.2.1333 18606688

[13] Rodrigues MM, Oliveira AC, Bellio M (2012) The Immune Response to *Trypanosoma cruzi*: Role of Toll-Like Receptors and Perspectives for Vaccine Development. J Parasitol Res 2012: 507874. 22496959 10.1155/2012/507874 PMC3306967

[14] Cardillo F, Voltarelli JC, Reed SG, Silva JS (1996) Regulation of *Trypanosoma cruzi* infection in mice by gamma interferon and interleukin 10: role of NK cells. Infect Immun 64(1), 128–134. 10.1128/iai.64.1.128-134.1996 PMC173737 8557330

[15] Aliberti JCS, Cardoso MAG, Martins GA, Gazzinelli RT, Vieira LQ, et al. (1986) IL-12 mediates resistance to *Trypanosoma cruzi* infection in mice and is produced by normal murine macrophages in response to live trypomastigote. Infect Immun 64(6), 1961–1967. 10.1128/iai.64.6.1961-1967.1996 PMC174023 8675294

[16] Ropert C, Gazzinelli RT (2004) Regulatory role of Toll-like receptor 2 during infection with *Trypanosoma cruzi*. J. Endotoxin Res 10(6), 425–430. 10.1179/096805104225006507 15588426

[17] Hunter CA, Ellis-Neyes LA, Slifer T, Kanaly S, Grunig G, et al. (1997) IL-10 is required to prevent immune hyperactivity during infection with *Trypanosoma cruzi*. J Immunol 158(7), 3311–3316. 9120288

[18] Cook DN, Pisetsky DS, Swartz DA (2004) Toll-like receptors in the pathogenesis of human disease. Nat Immunol 5(10), 975–979. 10.1038/ni1116 15454920

[19] O’Neill LA, Bryant CE, Doyle SL (2009) Therapeutic targeting of Toll-like receptors for infectious and inflammatory diseases and cancer. Pharmacol Rev 61(2), 177–197. 10.1124/pr.109.001073 PMC2846156 19474110

[20] Ford AQ, Smith E, Noben-Trauth N, Keegan AD (2009) Alternatively activated macrophages participate in the recruitment of eosinophils to the lung in a murine model of allergic lung inflammation. Suppl J Immunol 182, 79–82.

[21] Sieve AN, Meeks KD, Lee S, Berg RE (2010) A Novel Immunoregulatory Function for IL-23: Inhibition of IL-12 Dependent IFN-γ Production. Eur J Immunol 40(8), 2236–2247. 10.1002/eji.200939759 PMC3039303 20458705

[22] Trinchieri G, Sher A (2007) Cooperation of Toll-like receptor signals in innate immune defense. Nat Immun Rev 7(3), 179–190. 10.1038/nri2038 17318230

[23] Wang J, Hu Y, Deng WW, Sun B (2009) Negative Regulation of Toll-like receptor signaling pathway. Microbes Infect 11(3), 321–327. 10.1016/j.micinf.2008.12.011 19146978

[24] Lima ESL, Garcia I, Vicentelli M-H, Vassalli P, Minoprio P (2007) Evidence for a Protective Role of Tumor Necrosis Factor in the Acute Phase of *Trypanosoma cruzi* Infection in Mice. Infect Immun 65(2), 457–465. 10.1128/iai.65.2.457-465.1997 PMC174617 9009297

[25] Silva JS, Vespa GN, Cardoso MA, Aliberti JC, Cunha FQ (1995) Tumor necrosis factor alpha mediates resistance to *Trypanosoma cruzi* infection in mice by inducing nitric oxide production in infected gamma interferon-activated macrophages. Infect Immun 63(12), 4862–4867. 10.1128/iai.63.12.4862-4867.1995 PMC173696 7591147

[26] Silva GK, Gutierrez FR, Guedes PM, Horta CV, Cunha LD, et al. (2010) Cutting edge: Nucleotide-Binding Oligomerization domain1-dependent responses account for murine resistance against *Trypanosoma cruzi* infection. J Immunol 184(3), 1148–1152. 10.4049/jimmunol.0902254 20042586

[27] Pompei L, Jang S, Zamlynny B, Ravikumar S, McBride A, et al. (2007) Disparity in IL-12 release in dendritic cells and macrophages in response to *Mycobacterium tuberculosis* is due to use of distinct TLRs. J Immunol 178(8), 5192–5199. 10.4049/jimmunol.178.8.5192 17404302

[28] Hou B, Benson A, Kuzmich L, DeFranco AL, Yarovinsky F (2011) Critical coordination of innate immune defense against *Toxoplasma gondii* by dendritic cells responding via their Toll-like receptors. Proc Natl Acad Sci USA 108(1), 278–283. 10.1073/pnas.1011549108 PMC3017180 21173242

[29] Janssens S, Burns K, Tschopp J, Beyaert R (2002) Regulation of Interleukin-1 and lipopolysaccharide-induced NF-kappa B activation by alternative splicing of MyD88. Curr Biol 12, 467–471. 10.1016/s0960-9822(02)00712-1 11909531

[30] Kobayashi K, Hernandez LD, Galán JE, Janeway CAJr, Medzhitov R, et al.. (2002) IRAK-M is a negative regulator of Toll-like receptor signaling. Cell 110, 191–202. 10.1016/s0092-8674(02)00827-9 12150927

[31] Sengupta D, Koblansky A, Gaines J, Brown T, West AP, et al. (2010) Subversion of innate immune responses by *Brucella* through the targeted degradation of the TLR signaling adaptor Mal. J Immunol 184(2), 956–964. 10.4049/jimmunol.0902008 PMC3644118 20018612

[32] Franklin BS, Parroche P, Ataíde MA, Lauw F, Ropert C, et al.. (2009) Malaria primes the innate immune response due to interferon-gamma induced enhancement of toll-like receptor expression and function. Proc Natl Acad Sci USA 106(14), 5789–94. 10.1073/pnas.0809742106 PMC2657593 19297619

[33] Quesniaux V, Fremond C, Jacobs M, Parida S, Nicolle D, et al. (2004) Toll-like receptor pathways in the immune response to *Mycobacteria*. Microbes and Infect 6(10), 946–959. 10.1016/j.micinf.2004.04.016 15310472

[34] Lai Y, Di Nardo A, Nakatsuji T, Leichtle A, Yang Y, et al.. (2009) Commensal bacteria regulate Toll-like receptor 3- dependent inflammation after skin injury. Nat Med 15(12), 1377–82. 10.1038/nm.2062 PMC2880863 19966777

[35] Redecke V, Häcker H, Datta SK, Fermin A, Pitha PM, et al.. (2004) Cutting edge: activation of Toll-like receptor 2 induces a Th2 immune response and promotes experimental asthma. J Immunol 172(5), 2739–2743. 10.4049/jimmunol.172.5.2739 14978071

[36] Agrawal A, Dillon S, Denning TL, Pulendran B (2006) ERK1^−/−^ mice exhibit Th1 cell polarization and increased susceptibility to experimental autoimmune encephalomyelitis. J Immunol 176(10), 5788–5796. 10.4049/jimmunol.176.10.5788 16670284

[37] Dillon S, Agrawal A, Van Dyke T, Landreth G, McCauley L, et al.. (2004) Toll-like receptor 2 ligand stimulates Th2 responses in vivo, via induction of extracellular signal-regulated kinase mitogen-activated protein kinase and c-Fos in dendritic cells. J Immunol 172(8), 4733–4743. 10.4049/jimmunol.172.8.4733 15067049

[38] Wenink MH, Santegoets KCM, Broen JCA, van Bon L, Abdollahi-Roodsaz S, et al.. (2009) TLR2 promotes Th2/Th17 responses via TLR4 and TLR7/8 by abrogating the type I IFN amplification loop. J Immunol 183(11), 6960–6970. 10.4049/jimmunol.0900713 19915052

[39] Mellett M, Atzei P, Jackson R, O'Neill LA, Moynagh PN (2011) Mal mediates TLR-induced activation of CREB and expression of IL-10. J Immunol 186(8), 4925–4935. 10.4049/jimmunol.1002739 21398611

[40] Ivashkiv LB (2011) Inflammatory signaling in macrophages: transitions from acute to tolerant and alternative activation states. European J Immunol 41(9), 2477–2481. 10.1002/eji.201141783 PMC3264328 21952800

[41] Murray PJ, Wynn TA (2011) Obstacles and opportunities for understanding macrophage polarization. J Leuk Biol 89(4), 557–563. 10.1189/jlb.0710409 PMC3058818 21248152

